# The Association Between Using a Mobile Version of an Electronic Health Record and the Well-Being of Nurses: Cross-sectional Survey Study

**DOI:** 10.2196/28729

**Published:** 2021-07-06

**Authors:** Tarja Heponiemi, Anu-Marja Kaihlanen, Kia Gluschkoff, Kaija Saranto, Sari Nissinen, Elina Laukka, Tuulikki Vehko

**Affiliations:** 1 Department of Public Health and Welfare Finnish Institute for Health and Welfare Helsinki Finland; 2 Department of Health and Social Management University of Eastern Finland Kuopio Finland; 3 Research Unit of Nursing Science and Health Management University of Oulu Oulu Finland

**Keywords:** stress related to information systems, time pressure, usability, stress, health and social care

## Abstract

**Background:**

Mobile devices such as tablets and smartphones are increasingly being used in health care in many developed countries. Nurses form the largest group in health care that uses electronic health records (EHRs) and their mobile versions. Mobile devices are suggested to promote nurses’ workflow, constant updating of patient information, and improve the communication within the health care team. However, little is known about their effect on nurses’ well-being.

**Objective:**

This study aimed to examine the association between using a mobile version of the EHR and nurses’ perceived time pressure, stress related to information systems, and self-rated stress. Moreover, we examined whether mobile device use modifies the associations of EHR usability (ease of use and technical quality), experience in using EHRs, and number of systems in daily use with these well-being indicators.

**Methods:**

This was a cross-sectional population-based survey study among 3610 Finnish registered nurses gathered in 2020. The aforesaid associations were examined using analyses of covariance and logistic regression adjusted for age, gender, and employment sector (hospital, primary care, social service, and other).

**Results:**

Nurses who used the mobile version of their EHR had higher levels of time pressure (*F*_1,3537_=14.96, *P*<.001) and stress related to information systems (*F*_1,3537_=6.11, *P*=.01), compared with those who did not use mobile versions. Moreover, the interactions of mobile device use with experience in using EHRs (*F*_1,3581_=14.93, *P*<.001), ease of use (*F*_1,3577_=10.16, *P*=.001), and technical quality (*F*_1,3577_=6.45, *P*=.01) were significant for stress related to information systems. Inexperience in using EHRs, low levels of ease of use, and technical quality were associated with higher stress related to information systems and this association was more pronounced among those who used mobile devices. That is, the highest levels of stress related to information systems were perceived among those who used mobile devices as well as among inexperienced EHR users or those who perceived usability problems in their EHRs.

**Conclusions:**

According to our results, it seems that at present mobile device use is not beneficial for the nurses’ well-being. In addition, mobile device use seems to intensify the negative effects of usability issues related to EHRs. In particular, inexperienced users of EHRs seem to be at a disadvantage when using mobile devices. Thus, we suggest that EHRs and their mobile versions should be improved such that they would be easier to use and would better support the nurses’ workflow (eg, improvements to problems related to small display, user interface, and data entry). Moreover, additional training on EHRs, their mobile versions, and workflow related to these should be provided to nurses.

## Introduction

Viewing electronic health records (EHRs) on mobile devices such as tablets and smartphones has increased lately and is becoming more common in the health care sector in many developed countries. Nurses form the largest group in health care that uses EHRs and their mobile versions [1]. A previous study reported that use of mobile versions of EHR is common in hospitals, especially during the handover period and ward hours [1]. Nurses have been found to use the mobile version of EHR mainly for viewing inpatient lists, nursing notes, alerts, and patients’ clinical data with high frequency [2].

Mobile device use while working presents many benefits for nurses, as it helps in their workflow and allows real-time updating of patient information [3]. Use of mobile apps in home care has been associated with the promotion of nurse–patient relationship (as it helps both nurses and patients to express their feelings) and reduction in workload and stress, but they are also related to disturbance in personal life among nurses [4]. However, mobile devices are reported to be useful in improving the quality of care and decreasing stress among nurses [5].

In addition, mobile devices are suggested to benefit health care professionals by increasing convenience, accuracy, efficiency, and productivity as well as improving clinical decision making [6]. A previous review noted that mobile devices improved patient documentation through more complete recording, fewer documentation errors, and increased efficiency [7]. Moreover, mobile devices saved time, gave earlier access to new information, and enhanced work patterns [7]. Mobile devices are also regularly used to ensure effective team work within a health care team [8].

However, some downsides also exist related to the use of mobile devices in health care. For example, the problems in overall architecture and user interface may lead to an increased number of input errors, loss of data, or decreased efficiency [9]. During electronic data collection, mobile devices have been found to increase the time of the data entry by twofold and increase the risk of typing errors and missing data compared with electronic data collection on laptops [10]. Moreover, small screens may make information retrieval tasks difficult and increase incorrect choices and scrolling activities [11].

The effect of using mobile versions of EHRs on the well-being of nurses remains unclear. Viewing and updating EHRs on mobile devices might help nurses in their daily work by giving them a chance to use EHR while simultaneously caring for patients. Thus, mobile use might help reduce workload and consequently nurses’ work-related stress as well. By contrast, mobile device use may also elicit stress and frustration due to the aforementioned problems (ie, difficult user interface, small screens, and challenges in data entry [9-11]). Previous studies have shown that the usability of EHRs is associated with nurses’ well-being [12,13], and thus it could be assumed that mobile use might also have an effect.

Previous studies have shown that usability problems of EHRs, need to use many different systems, and inexperience in using EHRs have been associated with high levels of stress, time pressure, and psychological distress among health care employees [12-16]. However, it is not known whether using EHR with mobile devices has a moderating effect on these associations.

In the light of these previous findings, this study aimed to examine the association between using a mobile version of EHR and perceived time pressure, stress related to information systems, and self-rated stress. Moreover, we examined whether mobile device use modifies the associations of EHR usability (ease of use and technical quality), experience in using EHRs, and number of systems in daily use with the well-being indicators (time pressure, stress related to information systems, and self-rated stress).

## Methods

### Sample

Data were collected in the spring of 2020 via the online survey Webropol [17]. The link to the survey was sent via email by the Finnish Nurses Association, The Union of Health and Social Care Professionals in Finland (Tehy), and the National Professional Association for the Interests of Experts and Managers in Health Care (TAJA) to their members under 65 years of age, which included 58,276/80,622 nurses, midwifes, and public health nurses (representing 72.29% of the eligible population) [18]. One reminder was sent to nonresponders to maximize survey responses. A more detailed description of the data collection has been presented previously [18]. Altogether, 10,094 registered nurses opened the link and 3912 responded. Of those who responded, 302 answered that they did not perceive themselves as fit to answer the questionnaire because they had not practiced as registered nurses for a long time. Thus, the final sample included 3610 respondents (n=3340 [92.52%] women) aged between 22 and 65, with a mean age of 45.7 (SD 11.0) [18]. The sample was representative of the eligible population regarding regionality and employment sector. Women were slightly overrepresented and those aged under 40 were slightly underrepresented [18]. Ethical approval for the study was provided by The Finnish Institute for Health and Welfare (THL/482/6.02.01/2020).

### Measures

*Time pressure* was measured with the mean of 2 items measuring how often (during the previous half-year period) a person had been distracted by, worried about, or stressed about (1) being in a constant hurry and time pressure coming from unfinished work tasks and (2) having too little time to do work properly. The items were rated on a 6-point scale ranging from 1 (*never*) to 6 (*constantly*). The scale’s reliability (Cronbach α) was .94 in the study sample. This measure has been widely used previously and associated, for example, with poor EHR usability among nurses [13].

Stress related to information systems was measured with the mean of 2 items (α=.74) framed into a single question that asked how often (during the previous half-year period) the respondent had been distracted by, worried about, or stressed about (1) constantly changing information systems and (2) difficult, poorly performing IT equipment/software. The answers were rated on a 6-point scale ranging from 1 (*never*) to 6 (*constantly*). This measure has previously been used to evaluate and associated with, for example, employees’ distress and EHR usability [14,19].

*Self-rated stress* was measured with the widely used single-item self-rated stress measure [20]: “Stress means a situation when a person feels tense, restless, nervous, or anxious, or is unable to sleep at night because his or her mind is troubled all the time. Do you feel that kind of stress these days”? Response options were not at all/just a little/to some extent/quite a lot/very much. For analyses, these were categorized as 0=low stress (not at all/just a little/to some extent) and 1=high stress (quite a lot/very much).

*Mobile device use* was measured by asking respondents whether they also used EHR with mobile devices (such as a smartphone or tablet) with the following answer options: (1) yes (2) no, and (3) not possible to use mobile devices. The measure was coded as 0=no mobile device use (options 2 and 3) and 1=yes, uses mobile devices (option 1).

*Experience in using EHRs* was assessed by asking how experienced the respondent was in using an EHR (ie, as an EHR user) with a 5-point scale ranging from 1 (*beginner*) to 5 (*expert*). For analyses, this variable was coded as 0=*low experience* (answer options 1-3) and 1=*high experience* (answer options 4-5).

*The number of systems in daily use* was assessed by asking about the number of clinical systems that the responder needed to log into daily when working with patients. The response options were 0/1/2/3/4/5 or “more”/“my work does not include clinical work” (coded as *missing*). For analyses, the number of logins was coded as 1=1, 2=2, and 3=3 or more systems in daily use (5 respondents who answered that they had 0 systems in daily use were omitted from the analysis).

The usability measures *ease of use* and *technical quality* were measured with items derived from the validated National Usability-Focused Health Information System Scale (NuHISS) [21]. These measures assessed the usability of the current EHR system in use, not particularly the mobile version of the system. *Ease of use* included 3 items (α=.82) assessing the usability of key functionalities of the EHR system such as reading, documenting, and patient data retrieval (“The arrangement of fields and functions is logical on computer screen,” “Routine tasks can be performed in a straight forward manner without the need for extra steps using the system,” and “Terminology on the screen is clear and understandable [eg, titles and labels]”). *Technical quality* was measured with 4 items (α=.76) assessing reliability and safety aspects of the EHR system (“The systems are stable in terms of technical functionality [does not crash, no downtime],” “The system responds quickly to inputs,” “Faulty system function has caused a serious adverse event for the patient [reverse coded],” and “Faulty system function has nearly caused a serious adverse event for the patient [reverse coded]”). The answers were rated on a 5-point Likert scale ranging from 1 (*totally disagree*) to 5 (*totally agree*). The response options also included “Cannot answer,” which was coded as missing.

Besides, age, gender, and employment sector were asked in the survey. Employment sector was coded as 1=hospital, 2=primary care, 3=social services, and 4=other.

### Statistical Analysis

The associations of mobile use, experience in using EHRs, number of systems in daily use, ease of use, and technical quality with the time pressure and stress related to information systems were analyzed with analyses of covariance (in separate analyses for each dependent variable). The analyses were adjusted for age, gender, and employment sector. The analyses were conducted in 2 steps. In the first step (Model A), the analysis included mobile use, age, gender, and employment sector. In the second step (Model B) experience in using EHRs, number of systems in daily use, ease of use, and technical quality were added to the former model. Analyses regarding self-rated stress were conducted using logistic regression analyses with the same steps as mentioned above.

Moreover, we examined the interactions of mobile version use with experience in using EHRs, number of systems in daily use, ease of use, and technical quality for the dependent variables with analyses of covariance (for time pressure and SRIS) and logistic regression (for stress) adjusted for age, gender, and main effects (in separate analyses for each interaction and dependent variable).

## Results

### Demographics

The characteristics of the study population are presented in Table 1. A majority of the respondents were women and approximately half worked at hospitals. Using a mobile version of the EHR was not very common, and only 17.70% (639/3610) used mobile devices. Most often, mobile device was used in social services (101/445, 22.7%), after that in hospitals (377/1903, 19.81%) and other sectors (82/467, 17.6%), whereas it was less common in primary care (79/795, 9.9%).

**Table 1:** Social demographics of the study sample (N=3610^a^).

Characteristic		Value
**Gender, n (%)**		
	Women	3340 (92.52)
	Men	249 (6.90)
	Other (or did not want to report)	21 (0.58)
**Employment sector, n (%)**		
	Hospital	1903 (52.71)
	Primary care	795 (22.02)
	Social services	445 (12.33)
	Other	467 (12.94)
**Mobile device use, n (%)**		
	No	2971 (82.30)
	Yes	639 (17.70)
**Self-rated stress**		
	Low	2318 (64.41)
	High	1281 (35.59)
**Experience in using electronic health records, n (%)**		
	Low	1135 (31.44)
	High	2475 (68.56)
**Number of systems in daily use, n (%)**		
	1	1327 (37.16)
	2	1178 (32.99)
	3 or more	1066 (29.85)
Age^b^, mean (SD)		45.68 (10.97)
Stress related to information systems^c^, mean (SD)		3.70 (1.13)
Time pressure^c^, mean (SD)		4.54 (1.12)
Ease of use^d^, mean (SD)		3.01 (1.08)
Technical quality^d^, mean (SD)		3.25 (0.98)

^a^Because of missing information in some variables, n varies between 3571 and 3610.

^b^Ranged between 22 and 67.

^c^Ranged between 1 and 6.

^d^Ranged between 1 and 5.

### Main Effects

Age, gender, mobile use, number of systems in daily use, ease of use, and technical quality were associated with time pressure in the fully adjusted model (Model B; Table 2). Younger respondents, women, mobile device users, and those who had a higher number of systems in daily use perceived more time pressure. Higher levels of ease of use and technical quality were associated with less time pressure.

Age, gender, employment sector, mobile use, experience in using EHRs, number of systems in daily use, ease of use, and technical quality were all associated with stress related to information systems in the fully adjusted model (Model B). Older respondents, women, those working in hospitals, mobile device users, less experienced EHR users, and those who had a higher number of systems in daily use perceived more stress related to information systems. Higher levels of ease of use and technical quality were associated with less stress related to information systems.

**Table 2:** The associations of independent variables with stress related to information systems and time pressure (analysis of covariance).

Variable	Time pressure						Stress related to information systems					
	Model A			Model B			Model A			Model B		
	*F* test	*P* value	*F* test		*P* value	*F* test		*P* value	*F* test		*P* value	
Age	*F*_1,3583_=23.11	<.001	*F*_1,3537_=24.84		<.001	*F*_1,3583_=25.23		<.001	*F*_1,3537_=37.55		<.001	
Gender	*F*_2,3583_=9.13	<.001	*F*_2,3537_=8.25		<.001	*F*_2,3583_=8.51		<.001	*F*_2,3537_=6.00		.003	
Sector	*F*_3,3583_=2.31	.08	*F*_3,3537_=0.88		.45	*F*_3,3583_=43.93		<.001	*F*_3,3537_=24.38		<.001	
Mobile device use	*F*_1,3583_=15.87	<.001	*F*_1,3537_=14.96		<.001	*F*_1,3583_=7.41		.007	*F*_1,3537_=6.11		.01	
Experience			*F*_1,3537_=2.77		.10				*F*_1,3537_=17.31		<.001	
Number of systems in daily use			*F*_2,3537_=6.90		.001				*F*_2,3537_=43.17		<.001	
Ease of use			*F*_1,3537_=33.92		<.001				*F*_1,3537_=269.91		<.001	
Technical quality			*F*_1,3537_=62.83		<.001				*F*_1,3537_=311.82		<.001	
R^2^	0.016		0.069			0.043			0.301			

Age, number of systems in daily use, ease of use, and technical quality were associated with self-rated stress in the fully adjusted model (Model B; Table 3). Older respondents were less likely to have self-rated stress. Those who had 3 or more systems in daily use were 1.23 times more likely to have a high level of stress compared with those who had only 1 system in use. Higher levels of ease of use and technical quality were associated with lower likelihood of stress. Employment sector was significantly associated with stress in Model A (*P*=.02), but after adjusting for number of systems in daily use, experience in using EHRs, ease of use, and technical quality, the association was no longer significant (*P*=.17).

**Table 3:** The results of the logistic regression analysis for self-rated stress.^a^

Demographics			Model A					Model B				
			Odds ratio (95% CI)		*P* value		Odds ratio (95% CI)			*P* value		
Age			0.99 (0.98-0.99)		.001		0.99 (0.98-0.99)			.001		
**Gender**												
	Men	1				1						
	Women	1.33 (1.00-1.76)		.05		1.28 (0.96-1.71)			.10			
**Employment sector**					.02					.17		
	Hospital	1				1						
	Primary health care	0.90 (0.75-1.07)		.24		0.94 (0.78-1.12)			.47			
	Social care	0.87 (0.70-1.08)		.20		1.03 (0.82-1.29)			.82			
	Other	0.71 (0.57-0.89)		.003		0.78 (0.63-0.98)			.04			
**Mobile device use**												
	No	1				1						
	Yes, uses mobile device	1.05 (0.87-1.26)		.62		1.01 (0.84-1.22)			.91			
**Experience in using electronic health records**												
	Low					1						
	High					0.95 (0.81-1.11)			.49			
**Number of systems in daily use**										.04		
	1					1						
	2					1.00 (0.84-1.19)			.97			
	3 or more					1.23 (1.03-1.46)			.02			
Ease of use							0.76 (0.71-0.82)			<.001		
Technical quality							0.84 (0.78-0.91)			<.001		

^a^For continuous variables, the model odds ratio presented indicate the likelihood of passing from low stress to high stress, compared with 1 SD change in continuous independent variables.

### Interactions

We examined the interactions of mobile device use with experience in using EHRs, number of systems in daily use, ease of use, and technical quality for the dependent variables.

**Figure 1:**
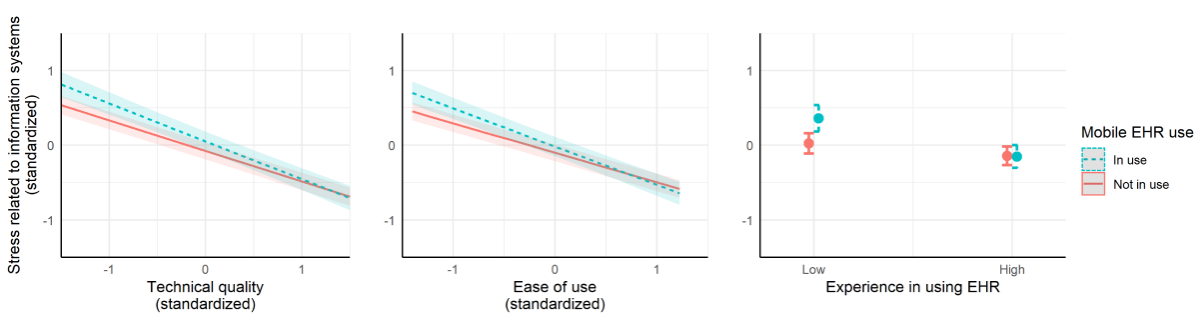
The associations of mobile device use with technical quality, ease of use, and experience in using EHRs for stress related to information systems. EHR: electronic health record.

The interaction between mobile device use and experience in using EHRs was significant for stress related to information systems (*F*_1,3581_=14.93, *P*<.001). As can be seen from Figure 1, the highest levels of stress related to information systems were reported by respondents who used mobile devices and had low experience in using EHRs. Moreover, the interaction between mobile device use and ease of use was significant for stress related to information systems (*F*_1,3577_=10.16, *P*=.001). The association between ease of use and stress related to information systems was more pronounced among those who used mobile devices and the highest levels of stress related to information systems was experienced among those who used mobile devices and perceived low levels of ease of use of their EHRs (Figure 1). Besides, the interaction between mobile device use and technical quality was significant for stress related to information systems (*F*_1,3577_=6.45, *P*=.01). Similar to ease of use, the association between technical quality and stress related to information systems was more pronounced among those who used mobile devices and the highest levels of stress related to information systems were experienced among those who used mobile devices and perceived low levels of technical quality of their EHRs (Figure 1). The interaction between mobile device use and number of systems in daily use was nonsignificant for stress related to information systems (*P*=.21).

## Discussion

### Principal Results

According to our results it seems that at present use of mobile versions of EHR is not beneficial to the nurses’ well-being. We found that use of mobile versions of EHR was associated with higher levels of time pressure and stress resulting from poorly functioning information systems.

Moreover, use of mobile versions of EHR intensified the association of inexperience and poor usability of EHR with stress related to information systems. More specifically, inexperienced EHR users who used mobile devices had higher levels of stress related to information systems than others. In addition, those who perceived low levels of ease of use or technical quality of their EHRs and used mobile devices had higher levels of stress related to information systems than others.

### Limitations

This was a cross-sectional survey study, and so we cannot draw any causal inferences. Moreover, we used self-reported measures which may have an effect here, given that if a respondent subjectively considers oneself being under stress, it is possible that it affects all responses, including those not related to stress. Therefore, it would be important for future studies to also include objective measures of stress when examining mobile versions. Using self-reported measures may also lead to a possibility of problems related to an inflation of the strengths of relationships and common method variance.

We adjusted our analyses for age, gender, and employment sector, but there may exist a possibility of residual confounding. Although the employment sector was adjusted for, it is possible that our results reflect higher work strain in places where mobile devices are used. In our study mobile device use was most common in social services and it is likely that, for example, in home care mobile devices are more often used and work strain can be high. This should be kept in mind when interpreting our results and future studies are needed on this topic. In addition, our study did not control for the technology used in the mobile version and given the possible wide variation in the usability of mobile versions this might be of importance. We also did categorize whether the mobile version used was a smartphone or a tablet, which has a big difference in size (and thus their user interface), and is likely to have affected the response of the nurses. Thus, these issues also need to be considered in the future.

Our sample was rather large (n=3610) which also allowed us to examine interactions. Our sample represented the eligible population regarding living region and employment sector, but included slightly more women and those aged over 40 [18]. Data were collected in the spring of 2020 (between March and April) at the time when the COVID-19 epidemic strengthened in Finland (with strict restrictions implemented mid-March). Therefore, only 1 reminder was sent to those who had not answered. This situation may influence the results and especially so in those hospitals that were most strongly affected.

Finland is one of the forerunners in the digitalization of health care [22] and provides a universal health care for all its residents. Therefore, caution should be exercised when generalizing our findings to other dissimilar countries.

### Comparison With Prior Work

Using EHR on the mobile device seems not to be beneficial for the well-being of nurses. We found that it was associated with high stress related to information systems and time pressure. Our finding is not congruent with previous findings suggesting that mobile device use would decrease nurses’ stress [4,5]. The discrepancy in the results may be due to different countries, methods, and samples studied. Chiang and Wang [4] performed a qualitative study including 17 community nurses from 6 home care facilities. Johansson et al [5] performed a quantitative study including 398 registered nurses and nursing students attending the undergraduate and graduate nursing programs, and thus their sample was smaller and included younger participants (mean age 34.7). Instead, we used a large (n=3610) population-based sample of registered nurses from different employment sectors. Moreover, the study by Johansson et al [5] focused more on advanced mobile devices themselves and on the capacity to locate information from the internet, whereas we focused solely on the mobile version of the EHR.

Although mobile devices are suggested to improve workflow, make continuous updating possible, and improve the communication within the health care team [3], there are many possible reasons as to why they could increase the sense of hurry and strain coming from viewing and updating EHRs on these devices. User interface has been suggested as one of the challenges in the deployment of clinical mobile apps [9]. Lack of visibility on mobile devices may lead to errors; interfaces are compact and cluttered with information which require full attention; and the change from focusing on real life toward a mobile device may pose problems [9]. Handheld devices have been found to double the data entry time and increase the risks of typing errors and missing data [10]. Small screen is also less effective and increases the number of scrolling activities [11]. Moreover, stability problems related to wireless networks may (negatively) affect nurses’ work when using mobile devices [23]. These challenges related to mobile devices may cause extra stress and time pressure to nurses. By contrast, it is also possible that mobile devices are more likely in use at those places where demands and hurry are at high levels.

According to our results, mobile device use intensifies the negative effects of usability problems related to EHRs on the stress that is caused by information systems. Ease of use and technical quality of the EHRs were also directly associated with all examined well-being indicators (ie, time pressure, stress related to information systems, and self-rated stress). Our findings correspond to previous studies showing that usability problems are associated with lower well-being among nurses [12,13] and physicians [14-16]. As mentioned above, mobile devices may have problems with user interface and data entry [9,10]. Thus, it is possible that difficulties in use or technical problems related to EHRs are also reflected in mobile devices causing extra stress and problems for nurses.

To promote the well-being of nurses, EHRs and their mobile versions should be improved, so they would be easy to use and would better support the workflow. Issues related to finance, hardware, communication, security, and user interface have been identified as main challenges regarding the implementation of clinical mobile apps [9]. Using interface that is already familiar to the user and the hierarchical organization of the information have been suggested as means to improve mobile apps [9]. It is important to accurately validate mobile device interfaces which are meant to be used in a clinical setting [24]. Nurses’ needs should be fully taken into consideration during the development of mobile versions [25]. For example, the functions that can increase performance and are associated with workflow are suggested to be of importance [2].

Our results suggest that inexperienced users of EHRs seem to be especially at risk when using mobile devices. A previous study has shown the importance of experience among physicians for managing SRISs and psychological distress levels [14]. Moreover, nurses’ low e-care competence has been associated with high time pressure and distress [13]. This calls for more training related to EHRs and their mobile versions. It would be important to provide adequate and systematic support and training for those who are just learning to use the system and who still have skills gaps (ie, focusing especially on new employees and those whose work environment is implementing new systems). For example, continuous educational programs focusing on enhancing nurses’ information technology literacy have been suggested [25]. Moreover, in-house information systems support and regular training on acquired information systems would be of importance and could also encourage positive attitudes toward technologies [26].

### Conclusions

According to our findings it seems that a mobile version of EHR is not beneficial for the well-being of nurses. Mobile device use was associated with nurses’ perceptions of higher levels of time pressure at work. Moreover, mobile device use was associated with higher stress resulting from poorly functioning and constantly changing information systems. In addition, mobile device use intensified the negative effects of inexperience in using EHRs and poor usability of EHRs on the stress related to information systems. Thus, it seems that at present mobile versions of EHRs need improvements to better support nurses’ workflow and well-being. Moreover, more training related to EHRs, their mobile versions, and workflow related to these should be provided to nurses.

It would be important to pay increasing attention to these issues, as nurses are at particular risk of experiencing additional stress and strain resulting from the need to use information systems in their work, and work strain, in turn, has been associated with a higher risk of disability [27]. A significant proportion of nurses’ working time is already spent on patient information systems and in addition to this, nurses must constantly learn to use a variety of new electronic services and platforms in their work, which have increased significantly as a result of the COVID-19 epidemic.

## Abbreviations

EHR: electronic health record

## References

[1] Lee Y, Park YR, Kim J, Kim JH, Kim WS, Lee J (2017). Usage Pattern Differences and Similarities of Mobile Electronic Medical Records Among Health Care Providers. JMIR Mhealth Uhealth.

[2] Kim S, Lee K, Hwang H, Yoo S (2016). Analysis of the factors influencing healthcare professionals' adoption of mobile electronic medical record (EMR) using the unified theory of acceptance and use of technology (UTAUT) in a tertiary hospital. BMC Med Inform Decis Mak.

[3] Schachner MB, Sommer JA, González Zulma A, Luna DR, Benítez Sonia E (2016). Evaluating the Feasibility of Using Mobile Devices for Nurse Documentation. Stud Health Technol Inform.

[4] Chiang K, Wang H (2016). Nurses' experiences of using a smart mobile device application to assist home care for patients with chronic disease: a qualitative study. J Clin Nurs.

[5] Johansson P, Petersson G, Saveman B, Nilsson G (2014). Using advanced mobile devices in nursing practice--the views of nurses and nursing students. Health Informatics J.

[6] Ventola CL (2014). Mobile devices and apps for health care professionals: uses and benefits. P T.

[7] Mickan S, Tilson JK, Atherton H, Roberts NW, Heneghan C (2013). Evidence of effectiveness of health care professionals using handheld computers: a scoping review of systematic reviews. J Med Internet Res.

[8] de Jong A, Donelle L, Kerr M (2020). Nurses' Use of Personal Smartphone Technology in the Workplace: Scoping Review. JMIR Mhealth Uhealth.

[9] Ehrler F, Wipfli R, Teodoro D, Sarrey E, Walesa M, Lovis C (2013). Challenges in the Implementation of a Mobile Application in Clinical Practice: Case Study in the Context of an Application that Manages the Daily Interventions of Nurses. JMIR Mhealth Uhealth.

[10] Haller G, Haller DM, Courvoisier DS, Lovis C (2009). Handheld vs. laptop computers for electronic data collection in clinical research: a crossover randomized trial. J Am Med Inform Assoc.

[11] Jones M, Marsden G, Mohd-Nasir N, Boone K, Buchanan G (1999). Improving Web interaction on small displays. Computer Networks.

[12] Kaihlanen A, Gluschkoff K, Hyppönen H, Kaipio J, Puttonen S, Vehko T, Saranto K, Karhe L, Heponiemi T (2020). The Associations of Electronic Health Record Usability and User Age With Stress and Cognitive Failures Among Finnish Registered Nurses: Cross-Sectional Study. JMIR Med Inform.

[13] Vehko T, Hyppönen H, Puttonen S, Kujala S, Ketola E, Tuukkanen J, Aalto A, Heponiemi T (2019). Experienced time pressure and stress: electronic health records usability and information technology competence play a role. BMC Med Inform Decis Mak.

[14] Heponiemi T, Kujala S, Vainiomäki Suvi, Vehko T, Lääveri Tinja, Vänskä Jukka, Ketola E, Puttonen S, Hyppönen Hannele (2019). Usability Factors Associated With Physicians' Distress and Information System-Related Stress: Cross-Sectional Survey. JMIR Med Inform.

[15] Vainiomäki S, Heponiemi T, Vänskä J, Hyppönen H (2020). Tailoring EHRs for Specific Working Environments Improves Work Well-Being of Physicians. Int J Environ Res Public Health.

[16] Vainiomäki Suvi, Aalto A, Lääveri Tinja, Sinervo T, Elovainio M, Mäntyselkä Pekka, Hyppönen Hannele (2017). Better Usability and Technical Stability Could Lead to Better Work-Related Well-Being among Physicians. Appl Clin Inform.

[17] Webropol.

[18] Saranto K, Kinnunen U, Koponen S, Kyytsönen M, Hyppönen H, Vehko T (2020). Nurses' competences in information management as well as experiences in health and social care information system support for daily practice. Finn J EHealth EWelfare.

[19] Heponiemi T, Hyppönen H, Kujala S, Aalto A, Vehko T, Vänskä J, Elovainio M (2018). Predictors of physicians' stress related to information systems: a nine-year follow-up survey study. BMC Health Serv Res.

[20] Elo A, Leppänen A, Jahkola A (2003). Validity of a single-item measure of stress symptoms. Scand J Work Environ Health.

[21] Hyppönen H, Kaipio J, Heponiemi T, Lääveri T, Aalto A, Vänskä J, Elovainio M (2019). Developing the National Usability-Focused Health Information System Scale for Physicians: Validation Study. J Med Internet Res.

[22] PwC (2014). European Hospital Survey: Benchmarking Deployment of e-Health Services (2012?2013): Composite Indicators on eHealth Deployment and on Availability and Use of eHealth Functionalities: Final Report. JRC Scientific and Policy Reports.

[23] Shen L, Zang X, Cong J (2018). Nurses' satisfaction with use of a personal digital assistants with a mobile nursing information system in China. Int J Nurs Pract.

[24] Ehrler F, Haller G, Sarrey E, Walesa M, Wipfli R, Lovis C (2015). Assessing the Usability of Six Data Entry Mobile Interfaces for Caregivers: A Randomized Trial. JMIR Hum Factors.

[25] Kuo K, Liu C, Ma C (2013). An investigation of the effect of nurses' technology readiness on the acceptance of mobile electronic medical record systems. BMC Med Inform Decis Mak.

[26] Ifinedo P (2016). The moderating effects of demographic and individual characteristics on nurses' acceptance of information systems: A canadian study. Int J Med Inform.

[27] Mäntyniemi A, Oksanen T, Salo P, Virtanen M, Sjösten N, Pentti J, Kivimäki M, Vahtera J (2012). Job strain and the risk of disability pension due to musculoskeletal disorders, depression or coronary heart disease: a prospective cohort study of 69,842 employees. Occup Environ Med.

